# In Vivo Fate of Diatom-Based Nanocarriers: Advances, Challenges, and Future Perspectives

**DOI:** 10.3390/ijms27114676

**Published:** 2026-05-22

**Authors:** Kshipra Naik, Luca De Stefano, Ilaria Rea

**Affiliations:** Institute of Applied Sciences and Intelligent Systems, Unit of Naples, National Research Council, 80131 Naples, Italy; kshipra.naik@na.isasi.cnr.it (K.N.); ilaria.rea@cnr.it (I.R.)

**Keywords:** diatom, nanocarrier, in vivo fate, biodistribution, intracellular fate, toxicity, biodegradation

## Abstract

Diatom nanotechnology offers significant potential for the development of innovative diatom-based nanocarriers for drug delivery and bioimaging, with promising implications for the treatment and diagnosis of diverse diseases. However, clinical translation of these nanocarriers remains limited due to an incomplete understanding of their in vivo fate. Current studies on the biodistribution, intracellular behavior, biodegradation, and clearance of diatom-based nanocarriers are inadequate and often lack systematic evaluation, leaving critical knowledge gaps. A comprehensive understanding of how these nanocarriers traverse biological barriers, interact with cellular components, and are ultimately eliminated from the body is essential for their rational design and safe clinical implementation. This perspective critically examines the in vivo fate of diatom-based nanocarriers, highlighting recent advances while identifying key challenges and unresolved questions. By integrating insights into their biodistribution, intracellular interactions, toxicological profile, biodegradation, and clearance mechanisms, this article provides a framework to guide the development of more effective and clinically relevant diatom-based nanoplatforms. Furthermore, it outlines future research directions and design strategies for next-generation nanoformulations, aiming to accelerate their translation from bench to the bedside.

## 1. Introduction

Nanotechnology has profoundly transformed the landscape of drug delivery and biomedical imaging/sensing by enabling the development of nanoscale platforms capable of improving therapeutic efficacy, reducing systemic toxicity, and enabling site-specific targeting [1]. A wide range of organic and inorganic nanocarriers including liposomes, polymeric nanoparticles, dendrimers, metallic nanoparticles, and silica-based systems have been extensively investigated for biomedical applications [2,3]. Among these, silica-based nanomaterials have attracted considerable attention due to their chemical stability, tunable porosity, high surface area, and versatile surface chemistry, which together enable multifunctionalization for efficient drug loading and controlled release, and bioimaging/biosensing applications [4,5]. In particular, diatom-derived biosilica has emerged as a unique and sustainable alternative to synthetic silica, offering distinctive structural and functional advantages.

Diatoms are a fascinating group of unicellular photosynthetic microalgae encased in intricate biosilica shells, known as frustules, which exhibit a Petri dish or hollow pill-box-like structure [6]. There are almost 250 living genera with over 200,000 estimated species of diatoms exhibiting colossal diversity [7]. The diverse shapes of frustules range from sparse skeletons of crisscrossing bars to barrels, pods, stars, triangles, and elaborate dishes that look like flying saucers [8]. However, only a handful of diatom species have been investigated and modified from the materials perspective until now. Diatom biosilica is biocompatible, biodegradable, nontoxic, chemically inert, and optically active, while also demonstrating thermal stability, high mechanical strength, a large surface area, and tunable surface chemistry, rendering them particularly attractive for biomedical applications [9]. Furthermore, diatoms are abundant, inexpensive, renewable, and environmentally friendly, aligning well with the principles of green nanotechnology. Their inherent biocompatibility, together with facile chemical modification strategies, enables the incorporation of therapeutic agents, imaging reporters, and targeting ligands, thus allowing the fabrication of multifunctional nanocarriers tailored for precision medicine. Notably, diatomite, obtained from fossilized diatoms, is a Food and Drug Administration (FDA)approved material for food and pharmaceutical production [10], further underscoring its safety and suitability for biomedical use.

Over the past decade, significant progress has been made in engineering diatom-based nanocarriers for controlled drug delivery, photothermal and photodynamic therapy, immunotherapy, diagnosis, and bioimaging [11,12]. Both intact frustules and frustule-derived micro- and nanoparticles have been functionalized with polymers, peptides, antibodies, and stimuli-responsive coatings to enhance circulation time, targeting specificity, and therapeutic performance. In vitro studies have demonstrated efficient cellular uptake, intracellular trafficking, and potent therapeutic efficacy of these nanocarriers across a broad spectrum of disease models, particularly in oncology [10,13]. Despite these promising advances, the clinical translation of diatom-based nanocarriers remains limited, primarily due to an incomplete understanding of a critical factor, the in vivo fate before and after the cargo delivery. The efficacy and safety of diatom-based nanocarriers remain significantly hindered by both nanocarrier-specific and physiological barriers. The nanocarrier-specific barriers include suboptimal physicochemical attributes (size, shape, and surface charge) and biological constraints such as immunogenicity, short plasma half-life, limited tissue penetration, and inefficient barrier crossing. These challenges are further compounded by physiological and pathological barriers, such as hepatic metabolism, renal filtration, immune system clearance, and various organ- and tissue-specific obstructions. These processes critically influence circulation time, biodistribution, long-term accumulation and toxicity in target tissues, intracellular trafficking, and ultimately elimination. Therefore, innovative solutions are urgently needed to develop diatom-based nanocarriers with optimized physicochemical and biological properties.

A fundamental requirement for the rational design and safe clinical implementation of any nanocarrier system is a comprehensive understanding of its behavior within complex biological environments [1,3,14]. For diatom-based nanocarriers, whose physicochemical properties including size, shape, porosity, morphology, surface chemistry, biocompatibility, and biodegradability differ substantially from conventional synthetic nanoparticles, these interactions are particularly complex and remain insufficiently characterized [15,16,17,18]. Diatom-based nanocarriers stand apart from conventional synthetic nanoparticles due to their intrinsic biogenic origin and hierarchically ordered architecture, exhibiting species-specific, three-dimensional porous structure with pore sizes spanning from nano- to microscale. Particularly, their broad size distributions, irregular and anisotropic geometries, and diverse surface chemistry, further complicates the prediction of their biological behavior [10,11]. Furthermore, they exhibit an amorphous nanopatterned surface topography that is specific to species [19]. These unique properties of diatom biosilica are attributed to many years of evolutionary selection and genetic control and hence cannot be replicated through synthetic means [20]. Therefore, it would be interesting to investigate the role of irregular and anisotropic geometry, surface topography and chemical cues on in vivo cellular interactions and biodegradation. Although recent investigations have begun to explore the in vivo fate of these nanocarriers, substantial disparities exist in their biodegradation and clearance kinetics across experimental media, cellular systems, and living organisms. Concerns regarding biodistribution become significantly more prominent at the level of whole organisms [21].

While numerous studies have explored the use of diatom-based nanocarriers for the delivery of therapeutics, diagnostic agents, and imaging probes, only a limited number of systematic investigations have focused on their in vivo fate. Existing studies on the in vivo fate of diatom-based nanocarriers are relatively sparse and often limited in scope, employing diverse testing models, administration routes, labeling strategies, and analytical techniques (Table 1). Consequently, reported outcomes vary widely, making direct comparison difficult and hindering the establishment of generalizable design principles [17,18]. Moreover, critical aspects such as long-term biodistribution, intracellular fate, immune interactions, potential toxicity, and biodegradation pathways remain poorly understood. These knowledge gaps represent major barriers to regulatory approval and clinical translation, underscoring the urgent need for systematic and mechanistic investigations [15,16,17,18].

Similarly, several reviews [15,16,22,23,24,25,26] have summarized recent advancements in diatom-based nanocarriers; however, they predominantly emphasize therapeutic, diagnostic, and theranostic applications while overlooking the crucial aspect of in vivo fate. This perspective article critically examines the current state of knowledge regarding the in vivo fate of diatom-based nanocarriers, focusing on biodistribution, intracellular interactions, toxicity, biodegradation, and clearance mechanisms. Furthermore, we discuss emerging strategies and research directions to optimize nanocarrier design, enhance biocompatibility, improve targeting efficiency, and ensure safe clearance. By integrating recent advances with critical analysis of unresolved challenges, we systematically examine the key factors governing the in vivo behavior of diatom-based nanocarriers and establish a comprehensive framework for understanding their interactions with biological systems. By addressing this knowledge gap, we aim to draw focused attention to the in vivo fate of these nanocarriers while providing actionable insights and inspiration for future investigations. Through this integrative perspective, we seek to accelerate the development of next-generation diatom-based nanocarriers with improved therapeutic performance and translational potential.

**Table 1:** In vivo studies of diatom-based nanocarrier.

Study (Author, Year)	Animal Model	Nanocarrier Type	Target Disease	Administration Route	Key Findings	Safety/Toxicity Observations	Biodistribution	Intracellular Studies	Biodegradation and Clearance
(1) Delalat et al., 2015 [11]	Subcutaneous mouse xenograft	Genetically engineered diatom *Thalassiosira pseudonana* with specific antibodies.	Neuroblastoma and B-lymphoma	Intraperitoneal	Genetically engineered biosilica frustules for targeted delivery to tumor sites.	-	Observed in liver and kidney but not in other vital organs.	Uptake by macrophages in reticuloendothelial system.	Accumulated and biodegraded in tumor site.
(2) Tramontano et al., 2023 [27]	In vitro	Gelatin-coated diatomite nanoparticles modified with anti-L1-CAM antibody and encapsulated in enteric polymer.	Colorectal cancer	Oral	Combination of local drug release and active targeting enhances the effect of delivered galunisertib.	No in vitro cytotoxicity	-	Active targeting approach enhanced the uptake.	Enteric polymer protected gelatin coverage from enzymatic biodegradation in stomach.
(3) Terracciano et al., 2015 [10]	In vitro	PEG and CPP-functionalized diatomite nanoparticles.	Breast cancer	-	PEG and CPP strongly reduced the cytotoxicity.	Hemocompatibility	-	CPP considerably increased the cellular uptake.	-
(4) Managò et al., 2018 [28]	In vitro	Diatomite nanoparticles conjugated with a non-targeting siRNA.	Human lung epidermoid Carcinoma	-	Raman imaging allowed a direct and label-free visualization of cellular uptake.	No cell toxicity	-	Particles located in endocytic vesicles in the perinuclear region.	Longer incubation times needed to observe exocytosis/dissolution.
(5) Adamis et al., 2000 [29]	Sprague-Dawley rats	Diatomaceous earth	Pulmonary toxicity	Intratracheal	Diatomaceous earth produced acute/subacute inflammation.	Caused higher than 90% haemolysis	-	-	-
(6) Bertke 1964 [30]	Weanling white rats of the Wistar strain	Diatomaceous earth	Subacute toxicity	Oral	Diatomaceous earth from fresh water less toxic than marine origin.	Histologic sections showed no damage.	-	-	-
(7) Hilding et al., 1981 [31]	Sprague-Dawley rats	Diatomaceous earth	Carcinogenicity	Oral	Diatomaceous earth had no demonstrable carcinogenic effect.	Autopsy studies revealed no significant increase in the incidence of malignant tumors.	-	-	-
(8) Janićijević et al., 2014 [32]	NMRI HAN mice	Peruvian food grade diatomite modified with aluminum sulfate.	Acute toxicity	Oral	Application of modified diatomite as a potential excipient.	Did not cause any kind of toxicological reaction or death.	-	-	-
(9) Lewin 1961 [33]	In vitro	Diatom *Navicula pelliculosa* (Breb.) Hilse	Dissolution	-	Recent diatom silica dissolves readily whereas fossil diatomite dissolves very slowly.	-	-	-	Adsorbed inorganic cations decrease the rate of dissolution of diatomaceous. Silica
(10) Maeda et al., 1986 [34]	Hartley-Duncan guinea pig	Flux-calcined diatomaceous earth	Acute and long-term pulmonary reaction	Intratracheal	Mild diffuse fibrosis first observed at 6 months and was still present at 15 months.	No significant cytotoxicity in vitro.	-	Phagocytosis was frequently observed in the alveolar spaces.	-
(11) Todd et al., 2014 [35]	4T1 tumor-bearing mice	Raw food grade diatoms tagged with iron oxide nanoparticles	Tumors	Intravenous	First example of in vivo translation of diatoms attracted to tumors by magnetic guidance.	Good in vitro biocompatibility; no abnormalities were observed in the animals.	Undesired particle accumulation in the lung.	-	Complete biodegradation takes longer time due to large size.
(12) Terracciano et al., 2019 [36]	*Hydra vulgaris*	Diatomite Nanoparticles	In vivo toxicity	-	Integration of multiple approaches at whole animal, cell, and molecular levels.	No effect on morphology, growth rate, and genetic analysis confirming their biosafety.	-	Cellular uptake observed in case of CPP-modified diatomite nanoparticles.	-
(13) Pratt 1983 [37]	Guinea pigs	Crystalline and Amorphous diatomaceous earth	In vivo Inhalation	Inhalation	Silicious dust that produces cell damage cleared more effectively from the lung.	Did not examine the acute morphological changes induced by diatomaceous earth.	Crystalline silica reached only 68 mg per lung, while amorphous silica was 120 mg.	-	-

## 2. Types of Diatom-Based Nanocarriers

Diatom-based nanocarriers can be classified into two types based on their origin, namely living biosilica obtained from cultured diatoms, and diatomaceous earth (diatomite) derived from fossils (Table 2). Living biosilica is produced by large-scale cultivation of diatoms in photobioreactors under controlled conditions, followed by extraction through purification processes. Whereas diatom skeletal remains accumulate as siliceous sedimentary deposits on ocean and lake beds, giving rise to diatomaceous earth derived from both freshwater and marine diatoms. Furthermore, the physical properties of diatomaceous earth, such as its amorphous or crystalline form, significantly influences its safety and efficacy. Natural diatomite is inherently amorphous (non-crystalline); however, heating (calcination) transforms it into crystalline forms, predominantly cristobalite and, to a lesser extent, tridymite. These crystalline forms have been reported to exhibit greater cytotoxicity and pose higher health risks than commonly encountered quartz. In contrast, the amorphous form has minimal or no fibrogenic properties but could become increasingly hazardous through industrial processing [34]. Processing involves treating raw amorphous diatomaceous earth at approximately 1000 °C through calcination or in the presence of a fluxing agent, such as sodium carbonate, in a process known as flux-calcination [38]. Therefore, in case of diatom-based nanocarriers, high-temperature treatment should thus be avoided. Moreover, diatom-based nanocarriers can be fabricated either as intact biosilica frustules or as micro- and nanoparticles derived from the frustule structures.

## 3. Structural Comparison of Diatom Biosilica and Mesoporous Silica Nanoparticles

**Figure 1:**
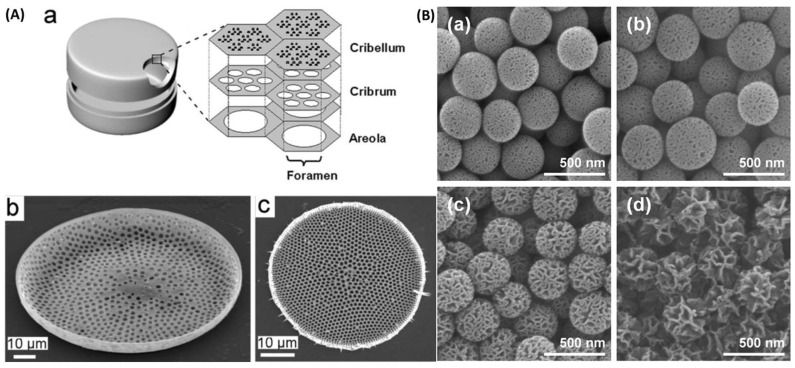
(**A**) (**a**) Schematic of a centric diatom frustule with a cross-sectional profile of the silica wall. Scanning electron microscopy images of cleaned diatom frustules, (**b**) *Coscinodiscus* sp. and (**c**) *T. eccentrica*. Reproduced with permission from [46]; published by [Royal Society of Chemistry, 2006]. (**B**) Scanning electron microscopy images of wrinkled silica nanoparticles with different interwrinkle distances controlled by the amount of added co-solvent (1-pentanol): (**a**) 0.16 mL, (**b**) 0.33 mL, (**c**) 0.65 mL, and (**d**) 1.30 mL. Reproduced under the terms of the Creative Commons CC BY license from [47]; published by [Springer Nature, 2016].

Diatom biosilica and mesoporous silica nanoparticles (MSNs) are both silica-based materials widely investigated for nanomedicine applications. However, their structural characteristics (Figure 1) differ fundamentally due to differences in their origins which critically influences their physicochemical behavior and performance in nanomedicine. Diatom biosilica is formed through biologically regulated silicification process [15], resulting in intricately patterned cell walls known as frustules. These structures are composed of amorphous hydrated silica and exhibit hierarchical organization [20], spanning multiple length scales, from micrometer-sized valves to nanoscale pores arranged in highly ordered geometries [24]. At the microscale, frustules display species-specific architectures (e.g., centric or pennate symmetry) characterized by periodic arrays of areolae, cribra, and interconnected pore channels [6]. These features generate a complex network of macro- (>50 nm), meso- (2–50 nm), and nanopores (<2 nm), yielding high permeability and specific surface areas typically up to 200 m^2^ g^−1^, depending on purification and post-treatment conditions [13]. This multiscale porosity facilitates high payload capacity and efficient mass transport, but the pore size distribution and geometry are inherently heterogeneous across species.

In contrast, MSNs are engineered using chemical synthesis processes that allow precise control over particle size, shape, and pore architecture [4]. They typically display uniform spherical or rod-like morphologies with well-defined, ordered mesoporous networks, often arranged in hexagonal or cubic symmetry [39]. These materials exhibit narrow pore size distributions typically within 2–50 nm and high specific surface areas in the range of ~600–1000 m^2^ g^−1^ [4,40]. The pore diameter, wall thickness, and particle dimensions can be tuned systematically during synthesis, enabling reproducible structural parameters and predictable diffusion pathways. Moreover, in comparison to diatom biosilica, MSNs lack intrinsic macroporosity and require an additional step of templating to achieve it.

The hierarchical complexity of diatom frustules provides structural robustness and large internal void volumes for loading bulky therapeutic agents and enabling their sustained release. In addition, diatom biosilica exhibits unique optical and photonic properties, arising from its periodic nanostructure, which can be leveraged for bioimaging and biosensing applications. However, the biological variability and rigid microscale dimensions of frustules (unless downsized to nanoparticles) can limit their ability to navigate biological barriers. Conversely, the smaller size and structural uniformity of MSNs support enhanced cellular uptake, controlled biodegradation, and integration into multifunctional nanomedicine platforms. Overall, naturally derived diatom biosilica offers a cost-effective and sustainable alternative, whereas MSNs are highly scalable but rely on chemically intensive synthesis routes. Diatom biosilica typically exhibits slower, more variable, and less predictable degradation profiles, in contrast to MSNs, whose degradation kinetics can be precisely tuned.

Diatom biosilica remains an emerging material in nanomedicine due to limited clinical evidence. While MSNs are considered the current benchmark in silica-based nanomedicine, being extensively studied and more translationally advanced, with certain systems (e.g., Cornell dots and Aurolase) having progressed to clinical evaluation [41,42]. In contrast to MSNs, whose in vivo fate, including biodistribution, intracellular uptake, biodegradation, and clearance, has been extensively characterized [4,5,39,43,44,45], diatom-based nanocarriers remain poorly understood in biological systems. Critical in vivo aspects of diatom-based nanocarriers such as long-term biodistribution, intracellular uptake, biodegradation kinetics, protein corona formation, and immune interactions are still largely unexplored. There is only one study demonstrating the complete in vivo biodistribution of genetically engineered diatom biosilica [11]. Limited understanding of the in vivo fate of diatom-based nanocarriers, due to lack of systematic studies and standardized characterization compared to well-studied MSNs, remains a major barrier to their clinical translation. In this perspective, design principles for diatom-based nanocarriers are extrapolated from MSN studies particularly with respect to established relationships between particle size, morphology, surface charge, and biological interactions, only when system-specific data are unavailable. This approach implicitly assumes comparable physicochemical–biological correlations. While MSNs provide a useful framework, such extrapolations overlook key differences in structural complexity, surface heterogeneity, and biosilica composition, which may result in distinct biological behaviors. Therefore, while MSNs provide a useful conceptual starting point, their design rules should be treated as guidelines rather than transferable principles, and diatom biosilica must be evaluated within its own structure–function framework through systematic, material-specific in vivo studies.

## 4. Factors Influencing the In Vivo Fate of Diatom-Based Nanocarriers

Diatom-based nanocarriers are anticipated to provide their cargo with protection against degradation, enhanced cellular uptake, and facilitation of endosomal escape. The in vivo fate of diatom-based nanocarriers depends on several factors such as morphology, porosity and pore size, size and surface area, zeta potential and surface charge, protein corona formation, concentration, encapsulation/loading efficiency, stability, surface modification and coating, targeting agents, immune response, and administration route, all of which are essential for the success and delivery efficiency of cargo.

### 4.1. Morphology

Morphology describes the 3D shape/form, structure, and surface roughness. It also describes surface wettability (hydrophilicity/hydrophobicity) which is closely tied to surface roughness and topographical features. Diatoms reflect immense diversity with more than 200,000 estimated species [7] exhibiting diverse frustule shapes, ranging from sparse skeletons of crisscrossing bars to barrels, pods, stars, triangles, and disks [8]. Therefore, the morphology of diatom-based nanocarriers widely varies with the species diversity and in case of micro/nanoparticles prepared from diatoms it is influenced by their preparation method. Morphology of diatom-based nanocarriers has an impact on their biodistribution, cellular uptake, and toxicity and can be commonly assessed by transmission electron microscopy, scanning electron microscopy, atomic force microscopy, and X-ray crystallography analysis that provide valuable information about the inner structure, shape, and dimensions.

Molecular dynamics simulations show that spherocylindrical nanoparticles are internalized more efficiently than spheres due to their larger volume, despite similar kinetic barriers, while sharp-edged particles inhibit uptake [48]. Phase diagram models further reveal shape-dependent cellular uptake modes. The high-aspect ratio rods with rounded tips enter side-first, whereas low-aspect ratio rods with flat tips enter tip-first [49]. The impact of morphology on in vivo fate of diatom-based nanocarriers has not been studied except in a handful of studies on silica nanoparticles that can be extrapolated to diatom biosilica. An in vitro study reported that the irregular shape of diatomite nanoparticles did not induce cell toxicity [28]. In case of MSNs, spherical shape was reportedly preferred to other shapes due to easier fabrication and faster internalization [4,50]. A comparison of spherical, short-rod, and long-rod MSN morphologies with aspect ratios of 1, 1.75 and 5, respectively, revealed that an increase in aspect ratio decreases the in vivo biodegradation and clearance of MSNs [51]. Moreover, in comparison with spherical particles, elongated particles exhibited slower renal clearance with preferential accumulation in the spleen and presented a shorter blood half-life, whereas particles with smaller aspect ratios promoted higher hepatobiliary clearance [52,53]. Short-rod MSNs were preferentially sequestered in the liver, whereas long-rod MSNs accumulated in the spleen. Additionally, short-rod MSNs were cleared more rapidly than long-rod counterparts through both the excretion pathways [52]. Furthermore, silica nanoparticles were also found to induce shape-dependent renal damage including hemorrhage, vascular congestion, and renal tubular necrosis [44,51]. Conversely, another study showed that increasing the aspect ratio enhances cellular uptake and accumulation, with rod-shaped MSNs (aspect ratio 2.1–2.5) exhibiting superior tissue penetration. According to some studies rod-shaped nanocarriers offered key advantages over spherical ones, including prolonged circulation, reduced reticuloendothelial system clearance, enhanced bioavailability, greater protein corona formation, and cellular uptake [45,54,55,56]. These conflicting results across studies make it difficult to define an optimal nanocarrier shape [4,57].

Nanocarrier shape has also been reported to influence the level of intracellular reactive oxygen species (ROS). In A375 human melanoma cells, long rod-shaped MSNs induced higher intracellular ROS levels but showed the lowest endocytic uptake compared to short rods and spherical particles [43,58]. MSNs with greater aspect ratios entered the cells more rapidly and disrupted actin cytoskeleton of the cells to a greater extent than ones with lower aspect ratios [43,59]. Furthermore, modifying the shape of MSNs can also influence their immunotoxicity; for example, asymmetric head-tail structured MSNs exhibited distinct immunomodulatory effects in comparison to conventional symmetric spherical MSNs [43,60]. Animal studies showed that nanocarrier shape also influences clearance kinetics, with spherical particles being eliminated more rapidly than rod-shaped MSNs [43,61].

### 4.2. Porosity and Pore Size

Porosity and pore size are also important determinants of nanocarrier fate and can be measured using nitrogen adsorption–desorption and electron microscopy methods. The enhanced biodegradability of mesoporous silica compared to its nonporous counterpart has been reported. MSNs with 10 nm pores degraded faster than those of 5 nm pores due to enhanced diffusion kinetics, further dependent on the size, symmetry, surface wettability, and chemical functionalities of the pores [44]. Also, MSNs with larger pore sizes (>30 nm) were reported to induce less intracellular ROS in macrophages and lacked pro-inflammatory effects in both cell culture and animal models [62]. MSNs with cone shaped pores were reported to reduce macrophage-based inflammatory responses and exerted significant osteo-immunomodulatory effects in vivo [43,63].

### 4.3. Size and Surface Area

Size of diatom-based nanocarriers like morphology would depend on the species or preparation method used and significantly affects their stability, encapsulation efficiency, blood circulation time, biodistribution, clearance, and biological performance [64,65]. Smaller nanocarriers of about 20–200 nm show improved stability, reduced aggregation, higher encapsulation efficiency, and prolonged circulation by evading reticuloendothelial system uptake [65]. In contrast, nanocarriers of <5 nm in size are rapidly cleared, those >50 nm tend to accumulate non-specifically in the liver, and larger nanocarriers of >200 nm preferentially accumulate in the spleen [18].

Diatom frustules exhibit different sizes ranging from 2 μm to 2 mm due to their diverse and complex morphology [7], whereas the nanoparticles derived from diatom frustules can be easily prepared in the size range of 100–400 nm [66]. The techniques used to measure the nanocarrier size are dynamic light scattering and electron microscopy analysis. Reducing the size of the nanocarrier significantly enhances key functionalities, including the solubility of poorly soluble drugs, immune evasion, cellular permeability, and passive tumor targeting via the enhanced permeability and retention effect. In future studies, it is important to develop size-selection strategies to enrich small-sized diatom-based nanocarriers of ≤500 nm as they show higher cellular uptake and may yield improved pharmacokinetics [35,67]. Notably, in an in-depth study of colloidal silica nanoparticles of various diameters under static aqueous conditions, the surface area and not the diameter of nanoparticles was found to control the degradation [68]. Additionally, comparative studies of aggregated and colloidal mesoporous silica nanoparticles exhibited incomplete degradation of aggregates, further confirming the critical role of surface area [44,68].

### 4.4. Zeta Potential and Surface Charge

Zeta potential influences the nanocarrier circulation time and tissue uptake while surface charge determines their interaction with serum proteins, cellular membrane, endosomes, and immune cells. Zeta potential is measured using dynamic light scattering. The positively charged nanocarriers may pose higher toxicity risks due to increased internalization, interaction with cell membrane, lower stability, and stronger immune response in comparison to the neutral or negatively charged ones [65]. Therefore, it is necessary to evaluate a range of zeta potential values that would lead to different outcomes such as rapid elimination from the blood stream or tendency to circulate in blood, and increased or reduced uptake by reticuloendothelial system. In case of MSNs, surface charge was reported to critically influence the lipoprotein adsorption from blood stream and therefore governed the in vivo excretion of nanocarrier. It was demonstrated that highly positively charged MSNs (zeta potential +34 mV) undergo rapid hepatobiliary clearance and fecal elimination, whereas negatively charged MSNs (zeta potential −18 mV) remain sequestered in the liver, consistent with the charge-dependent adsorption of serum protein [44,69].

### 4.5. Protein Corona Formation

It is important to understand the dynamics of the growth of protein corona on the surface of diatom-based nanocarriers from the perspective of how they behave in vivo. The protein corona is a very selective layer of proteins and other biomolecules strongly adsorbed on nanomaterial surface [70]. Techniques such as dynamic light scattering in combination with isothermal titration calorimetry, Fourier-transform infrared spectroscopy, and fluorescence spectroscopy can be useful tools in studying protein corona [71]. The composition of this layer is affected by nanomaterial properties such as shape, size, and charge. The transferrin-functionalized 50 nm silica nanoparticles were reported to lose their receptor-targeting ability when placed in a biological medium due to the development of protein corona [72]. Therefore, the detailed investigation of protein corona on nanocarrier surfaces will enhance their translation potential.

### 4.6. Concentration

Concentration of nanocarriers is a crucial parameter that affects silica biodegradation, toxicity, and clearance. Higher concentration of nonporous silica nanoparticle was reported to reduce their biodegradation rate which is governed by solubility limits [73]. Porous silica exhibits a solubility of ~120 ppm (40 mg L^−1^) in water at neutral pH and ambient temperature, which can be modulated by solubilizing agents (e.g., amines, hydroxides), ionic strength, and protein concentration [44].

### 4.7. Encapsulation/Loading Efficiency

The intricate hierarchical pore structures within diatom biosilica, ranging from nanoscale to microscale dimensions, provide exceptional opportunities for optimizing drug loading capacities and engineering advanced drug release profiles. This inherent porosity enables the loading of therapeutic agents both on the surface and within the internal voids of the biosilica, significantly impacting their release kinetics [11,74]. Consequently, the encapsulation/loading efficiency of diatom-based nanocarriers is a critical parameter for evaluating their performance, as it directly quantifies the amount of therapeutic agent successfully loaded into the frustule architecture [75]. It is typically calculated by determining the ratio of the total drug initially introduced to the amount of unbound drug remaining after the encapsulation process, often expressed as a percentage [76]. The encapsulation/loading efficiency reflects the amount of drug loaded on nanocarrier that is protected from biodegradation and successfully delivered to target cells. Therefore, it is a key determinant of drug delivery performance, with higher encapsulation efficiency indicating a more effective system. Early studies on the use of unmodified diatom frustules for drug delivery demonstrated an encapsulation efficiency of 14–22 wt% and sustained drug release over two weeks [77]. Subsequently, organosilane-functionalized diatom biosilica microcapsules tuned drug loading (15–24 wt%) and release (6–15 days); hydrophilic groups increased loading and slowed release, while hydrophobic groups reduced loading and accelerated release [78].

Efficient encapsulation is paramount for economic viability and therapeutic performance, minimizing drug waste while preventing premature degradation and systemic toxicity from unencapsulated drugs [15]. For example, the encapsulation of galunisertib drug in a hybrid nanoplatform further covered with gelatin lowered the amount of drug required to inhibit the metastatic process in cancer cells, hence preventing adverse side effects [66].

### 4.8. Stability

The stability of diatom-based nanocarriers is a crucial determinant of their efficiency and safety in drug delivery, as it influences structural integrity, drug retention, and behavior in physiological environments. Since instability can hinder clinical translation, assessing it at the early stages of development is strongly recommended. Owing to its highly ordered architecture, diatom biosilica exhibits excellent mechanical strength (~1700 kN m/kg) and resistance to degradation under ambient conditions, ensuring optimal shelf life and protection of encapsulated therapeutics during handling, storage, and transport conditions [11,79,80]. However, in biological fluids, these nanocarriers may experience reduced colloidal stability due to aggregation driven by high surface energy and protein adsorption, which can affect their circulation time, cellular uptake, and biodistribution [81]. Therefore, the nanocarrier stability in blood, or any other physiological media is critical and should be primarily addressed. Surface modification with biomolecules or polymers such as polyethylene glycol (PEG) enhances the stability of diatom-based nanocarriers by improving dispersion, reducing aggregation, and preventing premature degradation [44]. The chemical stability of diatom-based nanocarrier formulations is also influenced by environmental conditions such as pH, ionic strength, temperature, humidity, etc. Hence, it is important to assess the use of buffers, cryoprotectants, and surfactants to augment their stability [65].

### 4.9. Surface Modification and Coating

Surface modification of diatom-based nanocarriers with various molecules can distinctly modulate their biological fate by either stabilizing the porous architecture or promoting pore collapse. Moreover, the type of surface functionalization strongly influences the stability and biodegradation of MSNs. Hybrid mesoporous organosilica exhibited faster biodegradation and lower accumulation than conventional inorganic MSNs [43]. Furthermore, the coating of nanocarriers with polymers such as PEG can affect the membrane permeability, biodistribution, and immune response. PEG coating was reported to reduce protein adsorption and opsonization, enhancing stability, limiting macrophage uptake and immune activation, and prolonging nanocarrier circulation and residence time [17]. While polyethyleneimine (PEI) coating accelerated hydrolytic biodegradation by buffering pH conditions favorable for silica hydrolysis [43]. PEG-coated MSNs exhibited an average blood half-life of approximately 100 min longer than unmodified MSNs, while carboxylated MSNs showed a reduction of about 45 min [43].

### 4.10. Targeting Agents

The targeting agent impacts the nanocarrier biodistribution by interacting with specific receptors, transporters, enzymes, and immune cells present on the target tissue or cell type [65]. Several studies have reported the critical role that surface properties of nanocarriers play in their in vivo fate [82,83,84]. In case of an improper surface modification, the nanocarriers will be phagocytosed leading to an undesirable immune reaction which will further increase their toxicity [36]. Therefore, customization of surface properties with targeting agents potentially improves drug delivery specificity and efficiency [17].

### 4.11. Immune Response

Immune response including the innate and adaptive types influences the nanocarrier biodistribution by opsonization, phagocytosis, cytokine production, and antibody formation [65]. Hence, the uptake by macrophages or evasion of immune recognition to extend blood circulation time further needs to be studied.

### 4.12. Administration Route

The administration route influences the initial exposure of diatom-based nanocarriers to the blood circulation and organs, giving rise to varied drug delivery patterns [65]. It strongly impacts their absorption, biodistribution, and biodegradation. In case of MSNs, after intratracheal instillation or aspiration, the lungs serve as the primary target, whereas following injection or oral intake, the liver and spleen are predominantly affected. Among the different exposure routes, intravenous injection is the least well tolerated, followed by subcutaneous and intramuscular administration. In contrast, oral delivery is generally well tolerated even at very high doses (e.g., 5000 mg kg^−1^) [43]. Local injections can elicit systemic immune responses whereas pulmonary route can bypass the blood component interaction and systemic effects [65]. However, no studies on their impacts on diatom-based nanocarriers have been carried out to assess and compare different routes such as intravenous, intradermal, intramuscular, pulmonary, and oral. Therefore, different routes of administration should be carefully assessed to overcome the physiological barriers depending on the therapeutic application and target tissue.

Several rational design strategies of diatom-based nanocarriers can enhance the fate of therapeutics loaded onto them and therefore should be studied in detailed manner. The advancements in rational design and manufacturing would offer promising benefits for enhancing in vivo performance of diatom-based nanocarriers. Overall, controlling the quality attributes in diatom-based nanocarriers such as morphology, porosity and pore size, size and surface area, zeta potential and surface charge, protein corona formation, concentration, encapsulation/loading efficiency, stability, surface modification and coating, targeting agents, immune response, and administration route are critical for their successful therapeutic and diagnostic applications. Therefore, an in-depth assessment of these characteristics would contribute to the development of high-quality diatom-based nanocarriers. Furthermore, successful translation of diatom-based nanocarriers requires careful control of these factors in biological or physiological fluids, as they may change leading to instability and failure. Therefore, the in vitro testing of these nanocarriers should be conducted in media that simulates the biological conditions to predict their accurate physiological behavior.

## 5. Biodistribution Studies

Nanocarriers should selectively accumulate at the target disease site, such as a tumor, rather than in healthy tissues for their successful medical applications. If a significant fraction of nanocarriers accumulate in tissues or organs without undergoing further biodegradation or excretion, it may induce cytotoxicity and adverse effects. To minimize toxicity and collateral damage, nanocarriers should either degrade in situ into truly noncytotoxic byproducts or be efficiently cleared from the body after fulfilling their diagnostic or therapeutic function [21]. Several strategies have been developed to direct the biodistribution of MSNs including surface coating with folate, histidine, urokinase plasminogen activator, antibodies, and carbohydrates such as mannose [43]. The in vivo tracking of diatom-based nanocarrier accumulation and clearance can be achieved through various analytical methods. Biodistribution analysis, which examines the distribution of drug-loaded nanocarriers throughout the body post-administration, can be performed using radiolabeling, luminescence or fluorescence imaging, bioimaging, microscopy, and organ perfusion techniques [65].

The effectiveness of in vivo drug delivery is highly dependent on the interactions between diatom-based nanocarriers and blood components. Plasma proteins can adsorb onto the nanocarrier surface, forming a protein corona composed of diverse biomolecules that modulate their physiological properties. This protein corona can significantly influence biodistribution, blood circulation time, cellular uptake, immune recognition, and stability. Consequently, the design of nanocarriers necessitates strategies to modulate or minimize undesirable blood interactions [65]. Currently there are no studies reporting the protein corona formation and its composition in case of diatom-based nanocarriers. The concept of a uniform protein corona governing biological identity has been well established for conventional nanocarriers. However, it is likely oversimplified for diatom-based nanocarriers, where hierarchical porosity can enable protein infiltration into internal pores. Therefore, we hypothesize that its multiscale pore architecture may enable not only surface adsorption but also internal trapping of proteins, leading to a hybrid protein corona that could alter immune recognition and circulation over time. The internalized protein corona could act as a reservoir, prolonging biological identity and altering immune recognition compared to conventional nanocarriers. Therefore, pore accessibility could be tuned to select protein infiltration or exclusion by using surface gating or coatings to regulate internal corona formation.

The tissue and organ distribution of diatom-based nanocarriers remains incompletely understood due to factors such as blood flow variations and differential receptor expression. Additionally, optimizing nanocarrier size is crucial for achieving appropriate vascular permeability. In general, nanoparticles larger than 150 nm tend to accumulate in the liver and spleen, whereas those smaller than 5 nm are rapidly eliminated by renal filtration [85]. Furthermore, nanoparticle shape affects flow dynamics, thereby influencing biodistribution across different organs. Surface charge is also critical; positively charged nanoparticles are rapidly sequestered by macrophages in lungs, liver, and spleen, while neutral or negative nanoparticles show prolonged circulation and reduced organ accumulation [86]. Thus, in vivo biodistribution is dictated by the interplay of these parameters [1].

**Figure 2:**
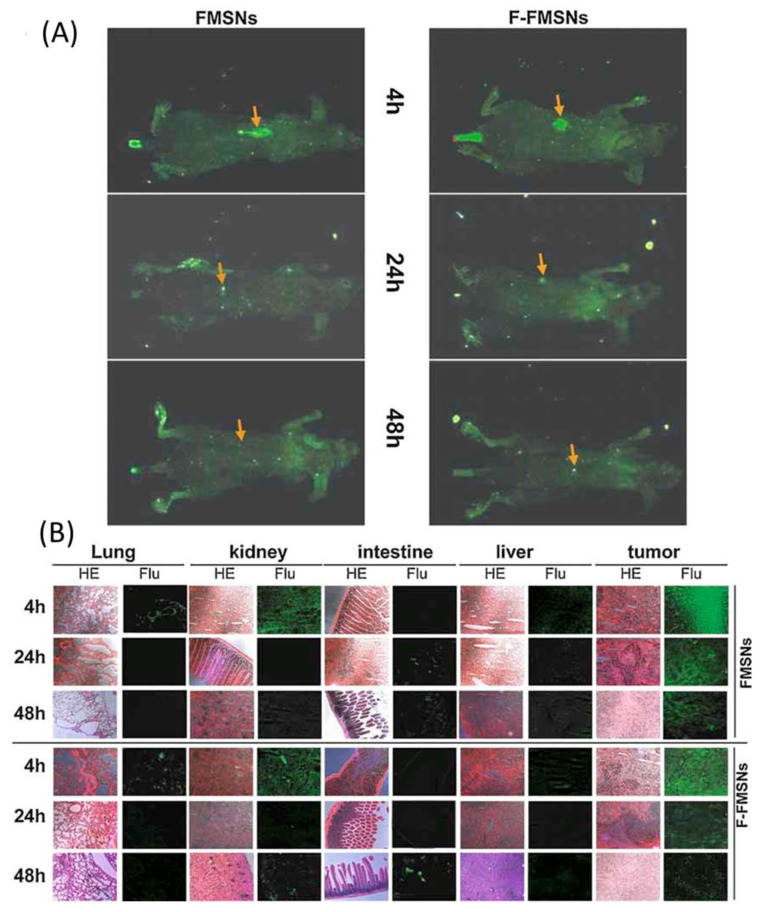
Biodistribution of fluorescent mesoporous silica nanoparticles (MSNs) in mice with xenograft tumors: (**A**) Representative fluorescence images of mice taken with a Maestro 2 in vivo imaging system. The yellow arrows show the subcutaneous tumors; (**B**) Representative tissue sections of mice are shown. All images shown here are at 100× magnification. HE = hematoxylin and eosin stain; Flu = fluorescence images taken with fluorescence microscope. Reproduced with permission from [61]; published by [Wiley, 2010].

Microscale biosilica derived from genetically engineered *Thalassiosira pseudonana*, expressing an antibody-binding domain on its surface for targeted cancer cell recognition, was employed as a drug nanocarrier. Biodistribution studies were conducted to assess potential side effects following intravenous administration of both targeted and non-targeted biosilica nanocarriers in nude mice, with daily observations over an eight-day period. The mice exhibited no signs of acute tissue damage, and optical microscopy analysis of tissue sections revealed no abnormalities in the brain, heart, kidney, liver, lungs, or tail. Biodegraded biosilica particles were observed to accumulate in the liver and kidneys, but not in the lungs or other vital organs, suggesting macrophage-mediated uptake (Table 1(1)) [11]. However, expanding the therapeutic potential of diatom-based nanocarriers requires improved biodistribution beyond hepatic accumulation. Notably, this remains the only study to date that has comprehensively reported the in vivo biodistribution of diatom-based nanocarriers across all organ systems in an animal model. The biodistribution studies of fluorescent MSNs in mice with xenograft tumors (Figure 2), employing in vivo imaging, fluorescence microscopy, and inductively coupled plasma mass spectrometry, demonstrated preferential accumulation of MSNs in tumors [61].

## 6. Intracellular Fate of Diatom-Based Nanocarriers

The primary mechanism of nanocarrier cellular internalization is endocytosis, which involves membrane invagination or ruffling, followed by the formation of intracellular endocytic vesicles. Depending on the type of specific or non-specific interactions that nanocarriers have with the cell surface, different endocytic mechanisms exist to mediate their entry into the cell. Endocytosis can be classified into two main types, viz. phagocytosis which is restricted to specialized immune cells, and pinocytosis which is the primary pathway in most cells. Pinocytosis comprises multiple distinct routes, including macropinocytosis, clathrin-mediated, caveolae-mediated, and clathrin/caveolin-independent pathways, which differ in vesicle characteristics and intracellular fate [87]. Following internalization, these nanocarriers are transported to endosomes, where they undergo biodegradation and are subsequently directed to lysosomes upon acidification. Therefore, endosomal escape is a critical step for effective drug delivery. However, the precise mechanisms governing this process in diatom-based nanocarriers remain largely unexplored and require further in-depth investigation.

The intracellular fate of nanocarriers is crucial for evaluating their cytotoxicity, as their persistence within cells significantly influences the effective drug dose. Once internalized by target cells, these nanocarriers induce cell death due to the pharmacological effects of the delivered drug. Consequently, understanding their in vivo fate, particularly after target cell death, is essential for assessing potential toxicity risks. To ensure the successful application of diatom-based nanocarriers in drug delivery, it is recommended to track their intracellular fate both before and after cell death. The cellular uptake and biodistribution of diatom-based nanocarriers have been analyzed using confocal fluorescence microscopy and transmission electron microscopy. However, these methods present limitations, including surface modifications due to fluorescent labeling and challenges in accurately identifying nanocarrier internalization.

**Figure 3:**
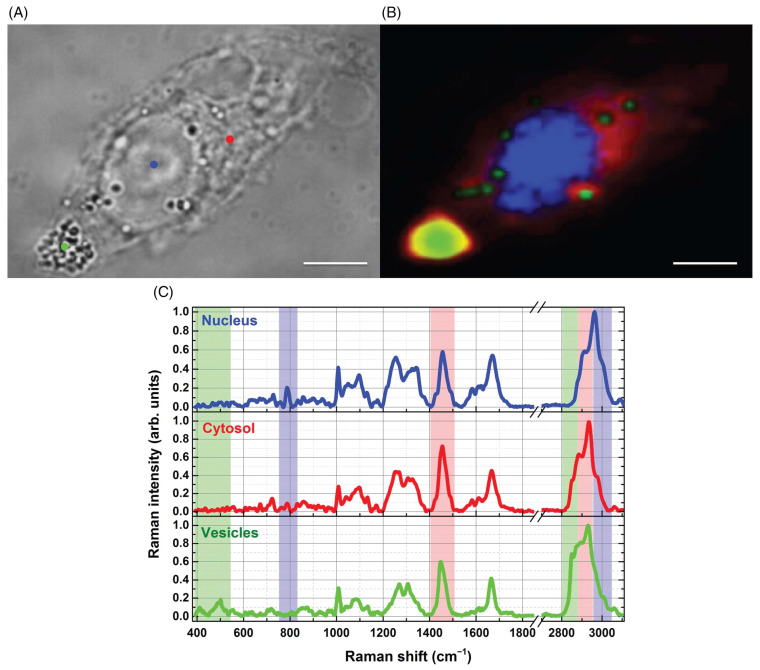
Raman imaging of diatomite nanoparticles (DNPs) uptake in H1355 cell: (**A**) optical image of the H1355 cell incubated for 24 h with DNPs. The dots indicate the location where the representative Raman spectra corresponding to nucleus, cytoplasm and vesicles were acquired. (**B**) Reconstructed Raman image of the selected cell by multivariate curve resolution method. The nucleus in blue and the cytoplasm in red are clearly visible. The lipid vesicles incorporating the DNPs are shown as green spots. (**C**) Representative Raman spectra of the nucleus (blue), cytoplasm (red) and vesicles with DNPs (green). Reproduced with permission from [28]; published by [Wiley, 2018].

To overcome these drawbacks, an in vitro study employed Raman microscopy to track the intracellular localization of diatomite nanoparticles (DNPs) over 72 h without the need for fluorescent labeling. Nanocarriers, ranging in size from 100 to 500 nm, are expected to slowly but extensively internalize via endocytosis. Therefore, DNPs of approximately 330 nm in diameter were analyzed for their uptake and cytotoxicity in H1355 lung cancer cells using Raman imaging (Figure 3). Raman bands characteristic of DNPs, and cellular components confirmed nanovector internalization and their co-localization within lipid environments. Cellular uptake kinetics revealed the presence of both internalized and membrane-associated DNPs at 6 h, followed by a saturation phase at 18 h, during which the nanoparticles were efficiently distributed within the cytosol. After 18 h, the percentage of internalized nanoparticles remained constant, with an increased accumulation near the nuclear periphery. Despite their intracellular persistence for 72 h, no nanoparticles were detected within the nuclear region, nor did they significantly affect cell viability or morphology, as confirmed by the MTT (3-[4,5-dimethylthiazol-2-yl]-2,5-diphenyl tetrazolium bromide) assay. However, extended incubation periods were suggested to further investigate the mechanisms of exocytosis and/or nanoparticle dissolution. Additionally, Raman microscopy findings were consistent with confocal fluorescence microscopy and photoluminescence data (Table 1(4)) [28].

It is essential to monitor the lysosomal degradation and cellular elimination rates of nanocarriers before their in vivo release upon cell death. Furthermore, the potential protective effects of polymer encapsulation on nanocarrier intracellular fate should be explored. However, no comprehensive studies have detailed the intracellular biodegradation and elimination processes of diatom-based nanocarriers. Additionally, strategies to enhance their intracellular uptake need to be developed and tested. One possible approach is modifying the final encapsulation layer, as it significantly influences cellular interactions, uptake efficiency, and nanocarrier internalization. For instance, a final layer composed of a positively charged polymer has been reported to enhance nanocarrier uptake [88].

## 7. Toxicological Studies

It is noteworthy that orally administered silica nanoparticles exhibit minimal toxicity, as silica has been classified by the FDA as “generally recognized as safe” for over 50 years, a status further demonstrated by 11 clinical trials and 2 clinical studies [45,89]. Nevertheless, there is a lack of clarity regarding the critical cellular mechanisms of diatomaceous earth toxicity and the particle characteristics that are directly responsible. Insights into particulate inhalation toxicity can be derived from in vitro cell culture studies, which provide mechanistic understanding while avoiding the cost and time constraints associated with in vivo inhalation models [90]. However, comprehensive in vivo studies are essential to assess long-term toxicity of diatom-based nanocarriers, as in vitro models fail to capture complex, integrated cellular and tissue interactions of whole-organism physiology.

### 7.1. Parameters Affecting Toxicity

The toxicity studies conducted previously [91,92,93] suggested crystalline silica to be more toxic as compared to the amorphous silica. However, as these studies mostly focused on crystallinity, neglecting other exposure parameters like size, geometry, surface area, volume, particle density, and composition, it is unclear if differences were due to crystalline silica content or other parameters [90]. An in vitro study using cultured Chinese hamster ovary cells compared natural amorphous and flux-calcined diatomaceous earth, pure crystalline silica, alpha-quartz, and cristobalite to determine whether toxicity depends on crystallinity or other particle properties. The qualitative results indicated a concentration-dependent response pattern consisting of particle uptake, induction of micro- and polynuclei, and reduction in cell proliferation with small decreases in viability. However, the toxicity did not correlate with crystallinity and natural diatomaceous earth that is primarily amorphous was found to be the most toxic of the silica dusts. Hence it was concluded that the in vitro toxicity is determined by a combination of density (number of particles/unit mass) and the proportion of particles having at least one dimension greater than 7.5 μm. Thus, in this study, the major mechanism of toxicity was cytostasis and cytokinesis which was dependent on particle number and particle size [90]. Another in vitro study also reported that natural diatomaceous earth was more potent than calcined form in inducting both hemolysis and the release of intracellular and lysosomal enzymes from macrophages [94]. Likewise, whether crystalline silica is carcinogenic to humans is still a debated issue in scientific literature.

Moreover, the diatomaceous earth deposits are variable in composition and diatom species and exhibit a wide range of physicochemical properties. An in vitro study addressed the variability of diatomaceous earth in terms of global source and processing technique using physicochemical characterization and haemolysis. Nineteen samples consisting of unprocessed, calcined and flux-calcined diatomaceous earth from around the globe were sourced and tested. Unlike previous reports, crystalline silica content alone was not an indicator of the potential toxicity of diatomaceous earth; however, particle size, surface area, morphology and composition were reported as contributing factors [38]. Several studies suggested that dust toxicity could be attributed to various cell type-specific cytotoxic mechanisms. An in vitro study exposed alveolar macrophages to either quartz or chrysotile asbestos and illustrated that particle surface chemistry and phagocytosis are important determinants of cellular response [95].

### 7.2. Hemotoxicity Studies

The hemolytic capacity and the surface silanol groups of amorphous silica are reported to increase with heat treatment up to 500 °C and further decrease with calcination from 500 to 1000 °C. A study demonstrated a direct relationship between the hemolytic capacity of silica and the quantity of surface silanol groups and hence reported amorphous silica to be 10-fold more toxic [96]. Another study reported an inverse relationship between average particle size and toxicity due to the increase in hemolytic capacity with surface area [97]. The hemocompatibility of diatom-based nanocarriers is extremely critical to avoid serious risks to humans. Additionally, hemolysis can also induce immunological reactions to inactivate the nanocarriers and eliminate them by macrophages. In vitro hemocompatibility and cytotoxicity study of amino, PEG and cell-penetrating peptide (CPP) functionalized diatomite nanocarriers for cancer drug delivery applications was reported. The hemotoxicity of amino-modified diatomite nanocarriers was higher than the diatomite modified with PEG and CPP when observed visually for the qualitative determination after 24 h. The percentage of hemolysis by spectrophotometric analysis also exhibited the same trend. Furthermore, scanning electron microscopy showed amino-modified diatomite nanocarriers altered red blood cell morphology, converting biconcave disks into shrunken forms, likely due to positively charged surface amine groups. However, no change in the red blood cell morphology was observed in case of PEG and CPP-modified diatomite nanocarriers. These nanocarriers were reported to be hemocompatible up to a concentration of 200 µg mL^−1^ for 48 h. Similarly, the functionalization with PEG and CPP was reported to reduce the cytotoxicity of diatomite nanocarriers when tested on breast cancer cells at 200 µg mL^−1^ for 24 h. Hence it was concluded that both the amino functionalization and charge density are significant contributing factors of toxicity (Table 1(3)) [10].

### 7.3. Free Radical Activity

An in vitro study found freshly fractured silica more toxic than aged silica, reducing cell viability and activating rat alveolar macrophages, indicating the role of fracture-generated surface radicals in toxicity [98]. In vitro free radical activity, cytotoxicity and morphological transformation of diatomaceous earth progressively heated in the laboratory at 900 and 1200 °C was evaluated in Syrian hamster embryo cells. Heated diatomite showed cell-transforming potential linked to generating hydroxyl radicals from its surface, indicating diatomaceous earth products may pose potential toxicity. Additionally, the finer fractions would exhibit a higher pulmonary toxicity when inhaled due to their higher surface reactivity and transforming potency. Therefore, the control of the heating process was suggested as the key factor for reducing the potential toxicity of manufactured diatomaceous earth products [99].

### 7.4. In Vivo Toxicity Studies

#### 7.4.1. Intravenous Route

**Figure 4:**
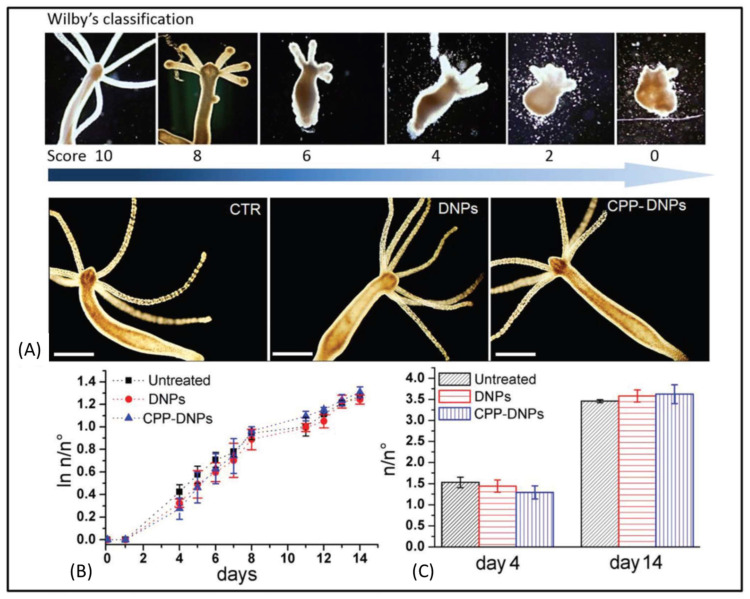
In vivo effects of DNPs on *Hydra* morphology and growth rate. (**A**) Upper panel: Wilby’s classification of *Hydra* morphological alterations due to exposition to a toxic environment. Lower panel: representative images of living *Hydra* polyps, untreated CONTROL (CTR) and treated with bare DNPs and CPP-DNPs up to 72 h. Scale bars, 500 μm. (**B**) The graph shows ln *n*/*n*0 values at each time point, where *n* is the total number of polyps and *n*0 is the number of founder ones. (**C**) The graph shows the *n*/*n*0 ratio standard deviation (s.d.) obtained from growth curves at day 4 and 14. Error bars represent s.d. (*n* = 3). Reproduced with permission from [36]; published by [Wiley, 2019].

The in vitro cytotoxicity of iron oxide nanoparticle-loaded diatomite tested at a concentration of 625 µg mL^−1^ in 4T1 murine breast cancer cells using MTT assay exhibited 80% viability after 24 h. But the in vivo intravenous administration (1.65 mg kg^−1^) in 4T1 tumor-bearing mice showed undesired lung accumulation due to entrapment of ~10 µm particles within narrow pulmonary capillaries [35]. However, the study did not report the toxicity of these nanocarriers in other organs of the animal. An integrated multilevel toxicological analysis of CPP-functionalized diatomite nanoparticles was carried out at the whole-living-organism level using a freshwater invertebrate, Hydra polyp, to assess complete in vivo biosafety portfolio (Figure 4). An exposure of high levels of 3.5 g L^−1^ of DNPs for 72 h demonstrated its biosafety at whole animal, cell, and molecular levels, including morphology, growth rate, apoptotic rate, and genetic analysis (Table 1(12)) [36].

#### 7.4.2. Oral Route

A study tested the in vivo carcinogenicity of ingested diatomaceous earth that was a constituent of water filters. No carcinogenic effects were observed in Sprague-Dawley rats fed with 20 mg diatomaceous earth daily for 840 days, mixed with cottage cheese containing 5 mg/g asbestos (Table 1(7)) [31]. An in vivo study in Wistar weanling white rats showed no histological tissue damage after feeding 1, 3, and 5% freshwater diatomaceous earth (~0.12 mm) for 90 days. Also, there was no significant increase in the percent of residual silica and no atrophy or hypertrophy observed in kidney, liver, and spleen due to the ingestion of diatomaceous earth (Table 1(6)) [30]. Aluminum compound functionalized diatomite with improved adsorption of diclofenac sodium drug was tested for its in vivo acute toxicity in mice. An oral dose of 2000 mg kg^−1^ diatomite sample was administered to the animals, and they were observed for symptoms of toxicity and death. The diatomite samples were reported to be safe drug delivery carriers as no toxicological reaction or death was observed during the period of 72 h (Table 1(8)) [32].

#### 7.4.3. Inhalation and Intratracheal Route

An in vivo inhalation study conducted a chemical analysis of lung tissue for its content of three different forms of inhaled silica viz. crystalline free silica (cristobalite), amorphous free silica (diatomaceous earth), and combined silica (volcanic glass) in guinea pigs. Fibrosis appeared at 15 months in animals exposed to crystalline silica, worsening by 21 months; in contrast, amorphous silica induced fibrosis only after 24 months and remained less severe. No fibrosis was observed in the animals exposed to the combined silica, although alveoli were reported to become heavily packed with phagocytic macrophages (Table 1(13)) [37]. Another study evaluated the acute and long-term pulmonary reaction in guinea pigs following intratracheal instillation of flux-calcined diatomaceous earth particles. Macrophages dominated particle phagocytosis, with minor neutrophil involvement, leading to mild diffuse fibrosis at 6 months that persisted through 15 months. (Table 1(10)) [34]. Similarly, a single intratracheal injection of 10 mg of unprocessed, amorphous diatomaceous earth exhibited potential toxicity causing acute/subacute inflammation in rats after 60 days (Table 1(5)) [29].

While the above studies discussed the in vivo toxicology of diatomaceous earth, no study reported the in vivo toxicity of living biosilica obtained by culture of diatom cells. Additionally, the in vivo and in vitro cytotoxicity results disagree due to biopersistence mechanisms and complex multicellular interactions in whole organisms that cannot be fully replicated in cell culture systems [90].

## 8. Dissolution and Biodegradability of Diatom-Based Nanocarriers

The dissolution of diatom-based nanocarriers in vitro provides crucial insights into their potential biodegradability in vivo. The dissolution rate of silica from the diatom walls of *Navicula pelliculosa* (Breb.) Hilse was investigated using a colorimetric method to assess the presence of a protective layer that could hinder dissolution. The study examined both living and fossilized diatoms from diverse origins, subjecting them to various pH levels, temperatures, salinities, exposure times, and different organic and inorganic cations. Results demonstrated that silica from living freshwater diatoms was more stable, likely due to an additional protective mechanism or physicochemical barrier. In contrast, amorphous silica from recently deceased diatoms dissolved rapidly, whereas fossilized diatoms exhibited significantly slower dissolution rates [100]. This reduced dissolution was attributed to a decrease in specific surface area over time, leading to fewer free silanol (Si-OH) groups and an overall decline in dissolution due to aging. Furthermore, adsorbed inorganic cations were found to slow the dissolution rate of diatomaceous silica. Variations in diatom wall thickness among species also influenced solubility, with differences in the surface-to-volume ratio playing a key role. These findings suggest that the solubility of diatom-based silica under natural conditions cannot be universally defined.

Additionally, the dissolution rate of silica frustules increased in media with higher pH, temperature, and solubility, but decreased over time. Among tested samples, silica from living diatom cells exhibited the slowest dissolution, followed by heat-killed cells, with cleaned silica walls dissolving the fastest (Table 1(9)) [33]. In a more applied study, galunisertib-loaded, gelatin-coated DNPs were developed and further modified with a targeting antibody and encapsulated in hydroxypropyl methylcellulose acetate succinate for oral colorectal cancer treatment. To evaluate their behavior in the gastrointestinal tract, dissolution studies were performed in simulated gastric fluid (pH 1.6, pepsin, 2 h), fasted-state simulated intestinal fluid (pH 5.5, trypsin, 2 h), and fasted-state simulated intestinal fluid (pH 8.0, trypsin, 2 h). Morphological analysis using transmission electron microscopy confirmed that the dissolution of the enteric polymer and gelatin enabled slow and sustained drug release at the target site. However, no studies specifically assessed the dissolution of DNPs, leaving their in vivo fate and safety unaddressed (Table 1(2)) [27].

Understanding the biodegradability of diatom-based nanocarriers in vivo is crucial for their biomedical applications, particularly in minimizing toxicity. A study investigated biodegradability of iron oxide nanoparticle-loaded diatomite in a body fluid mimic over one week, revealing fragmented nanostructures indicative of an ongoing degradation process. The prolonged time required for complete biodegradation was attributed to the relatively large particle size (~10 µm) (Table 1(11)) [35]. However, there are no comprehensive studies reporting the in vivo biodegradation studies of diatom-based nanocarriers. We hypothesize that diatom-based nanocarriers may undergo spatially heterogeneous biodegradation, where certain regions would degrade faster, leading to structural fragmentation. This could be attributed to the structural heterogeneity present in the diatom biosilica.

Nevertheless, Croissant et al. investigated the biodegradation of hierarchically porous MSNs and found that larger pore sizes accelerate degradation due to enhanced diffusion kinetics, which are governed by pore size and symmetry, surface wettability, and pore functionalization [101]. Furthermore, the condensation degree was reported as another fundamental parameter affecting the biodegradability of MSNs. The reactivity of silanol (Si-OH) bonds towards hydrolysis is much higher than that of siloxane (Si-O-Si) [102]. Therefore, a fully condensed silica network obtained by calcination post-synthesis exhibited very low dissolution rates [103]. Porous silica has a low degree of condensation and degrades more rapidly, whereas nonporous silica nanoparticles, with higher condensation from closely spaced silanol groups during synthesis, degrade more slowly [73]. Sphere shaped MSNs exhibited faster degradation in simulated media in comparison to the rod shaped due to the relatively larger external surface area [104]. The surface area played a key role in the dissolution kinetics of MSNs as it provided contact with the physiological fluid at the interfaces producing faster dissolution rates [68]. The degradation of MSNs was independent of their size when tested with diverse diameters in simulated fluids [68,105].

**Figure 5:**
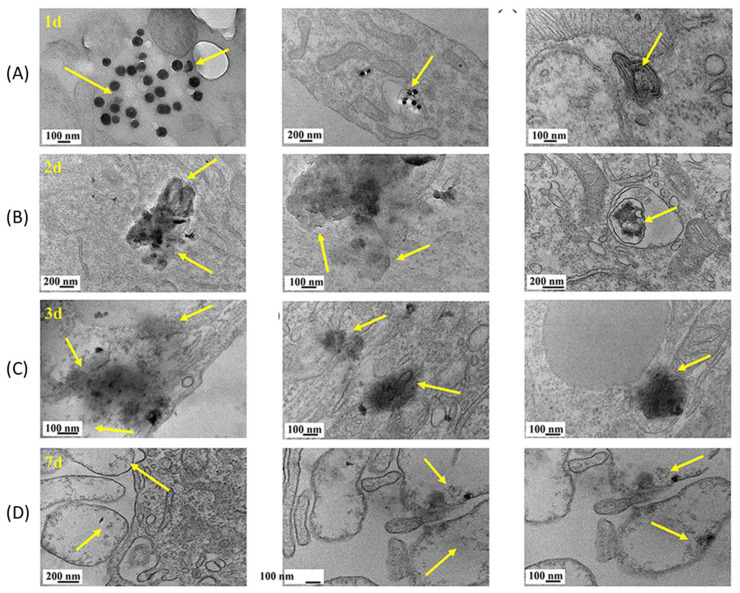
TEM images of cancer cells after coincubation with Mn-HMSNs to observe the intracellular biodegradation behavior at different time points (**A**) 1, (**B**) 2, (**C**) 3, and (**D**) 7 d, respectively. At initial stage (**A**) Mn-HMSNs are endocytosed by cancer cells and accumulate into the cytoplasm; at 48 h (**B**) after intracellular uptake the fast biodegradation of Mn-HMSNs is observed, as revealed by the cloudy morphology without a defined spherical structure; at day 3 (**C**) the biodegradation resulted in a significant fusion of degraded products; no significant material formulations could be found intracellularly after 7 days (**D**), demonstrating that the biodegraded species could be easily excreted out of the cells. Reproduced with permission from [106]; published by [American Chemical Society, 2016].

Furthermore, the effect of inorganic doping on MSN biodegradability was studied using diverse inorganic dopants including zirconium, calcium, iron oxide, manganese, and zinc. Zirconium doping inhibited biodegradation while calcium, iron oxide, and manganese exhibited faster dissolution and degradation rates. Manganese-doped hollow MSNs (designated as Mn-HMSNs) exhibited fast disintegration and biodegradation, further accelerating the breakage of Si-O-Si bonds within the framework (Figure 5) [106]. Zinc doping resulted in a more uniform and controlled biodegradation of the nanosystem [39]. Next, the effect of surface functionalization on MSN biodegradation was also investigated. The results demonstrated fastest biodegradation in phenyl-functionalized MSNs while PEGylation significantly inhibited it. The biodegradation of MSNs was also investigated in different dissolution media including simulated lung fluid (pH 7.40), simulated body fluid (pH 7.25), simulated gastric fluid (pH 1.60), and phosphate-buffered saline (pH 7.40) wherein simulated lung fluid exhibited fastest biodegradation rate due to the presence of organic acids. Also, the presence of proteins from fetal bovine serum were reported to accelerate the silica dissolution process. Moreover, the biodegradation of MSNs was also reported to depend on the dynamic/static condition of degradation media, amount administered, route of administration, and its site of action [39].

Subsequently the in vivo biodegradability of MSNs was studied in different animal models. In most cases, the size, shape, and surface functionalization of MSNs were identified as key parameters. The surface functionalization with polymeric coatings improved their stability and therefore increased the blood stream half-life. Administration route was also observed as an important parameter as when injected via subcutaneous injections, MSNs required additional time to enter and circulate in blood stream [107]. Given the lack of in vivo biodegradation studies of diatom-based nanocarriers, further comprehensive investigations are essential to determine their long-term fate and optimize their design for clinical applications.

## 9. Current Challenges and Future Perspectives

**Figure 6:**
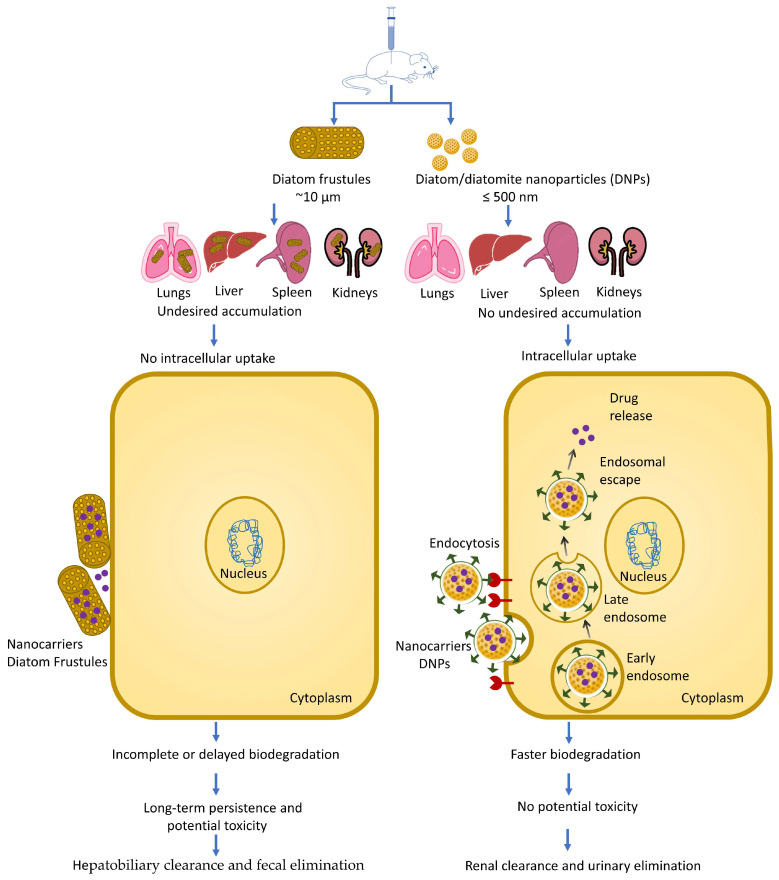
Schematic representation of the in vivo fate of diatom-based nanocarriers.

Diatom-based nanocarriers have emerged as promising platforms for drug delivery owing to their hierarchical porosity, high loading capacity, intrinsic biogenic origin, and multifunctional surface chemistry. Overall, these nanocarriers demonstrated good biocompatibility without any significant detrimental biological behaviors during preclinical investigations. However, their successful clinical translation is constrained by an incomplete understanding of their in vivo fate. The current understanding of their in vivo fate is shown in the scheme (Figure 6). A key challenge lies in the limited ability to quantitatively track these nanocarriers within complex biological environments. For many nanocarrier systems, the in vivo fate is inferred indirectly from drug signals rather than the nanocarrier itself. This is further complicated by the inherent physicochemical heterogeneity of diatom-derived structures, including variability in size, shape, and surface properties, which can lead to inconsistent biological interactions and unpredictable pharmacokinetics. Moreover, their relatively large and rigid architecture, unless engineered at the nanoscale, may limit their ability to traverse biological barriers and penetrate target tissues effectively. Upon systemic administration, these nanocarriers rapidly acquire a protein corona that redefines their biological identity, influencing immune recognition, cellular uptake, and organ accumulation. Similar to other silica-based systems, diatom-based nanocarriers tend to accumulate in the mononuclear phagocyte system, particularly in the lung, liver and spleen, raising concerns regarding their long-term persistence and potential toxicity, especially in case of incomplete or delayed biodegradation [35].

**Figure 7:**
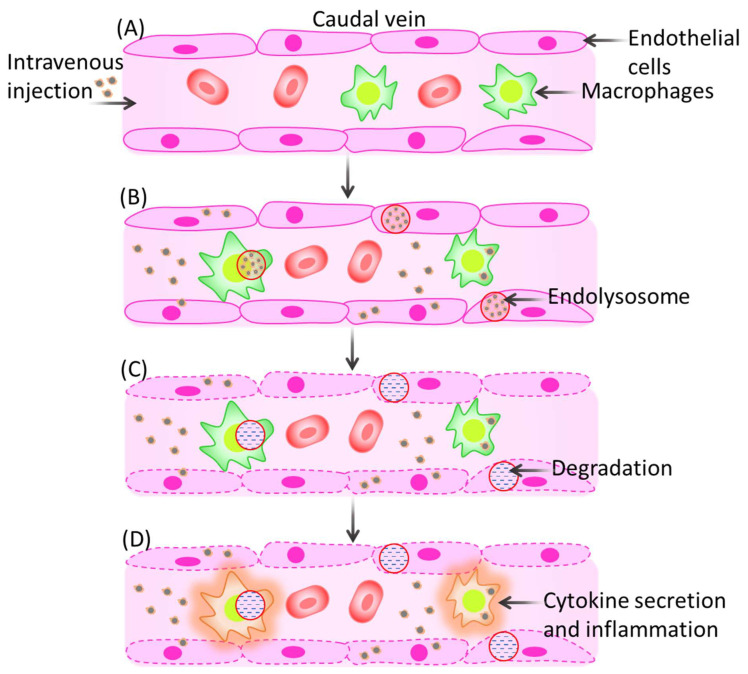
In vivo fate of diatom-based nanocarriers with a non-self biological identity: (**A**) intravenous injection of diatom-based nanocarriers with non-self protein corona; (**B**) diatom-based nanocarrier uptake and acidification in endolysosomes; (**C**) degradation of intracellular protein corona and loss of blood vessel integrity; (**D**) cytokine secretion and inflammation.

Accordingly, in light of these key challenges limiting their clinical translation, we highlight the following future research directions to address these barriers:

- The in vitro studies of diatom-based nanocarriers must include thorough physicochemical characterization before drawing any conclusions regarding the particular nanocarrier characteristic responsible for the toxic effects observed.
- It is critical to standardize the biosafety assessments in both in vitro and in vivo models to enable reliable comparisons for regulatory approval and clinical translation. Careful optimization of evaluation conditions and testing procedures is essential to minimize measurement errors and reduce variability. Establishing standardized biosafety testing protocols would improve reproducibility, decrease inconsistencies across studies, and enhance the overall quality of the results [43].
- During in vitro toxicological evaluation, it is necessary to consider cellular dosimetry. Particularly, in case of nanoparticles, the administered dose may differ substantially from the amount actually delivered to target cells. Additionally, direct nanoparticle and target cell contact is critical for accurate biocompatibility assessment. Therefore, quantifying the fraction of nanoparticles deposited on the target cell membrane and accounting for exposure time are essential for reliable dose–response analyses [43].
- Different research groups used diatom-based nanocarriers from different origins and species giving rise to wide diversity in physicochemical properties, making their toxicity comparison difficult. Going forward, researchers should deposit these data in a common and open archive which will be useful to study and compare their in vivo fates.
- The recently developed multifunctional diatom-based nanocarriers that integrate both diagnostic and therapeutic functionalities, have not been studied for their in vivo fate. These hybrid multifunctional nanodevices fall into a complex category, demanding extensive in vivo studies.
- Most published studies have examined the safety and efficacy of diatom-based nanocarriers in two-dimensional cell culture systems in vitro, which cannot reflect the in vivo extracellular environment. Therefore, it is suggested that the testing of diatom-based nanocarriers should be carried out using the three-dimensional immune-competent models to be able to mimic the complexity of in vivo physiological conditions.
- It is critically important to carefully consider the species compatibility during preclinical in vitro and in vivo testing for the development of diatom-based nanocarriers as it determines their fate. The mismatched species combinations during nanomedicine development can trigger adverse outcomes due to incompatible protein coronas (Figure 7). For example, it will be incorrect to use fetal bovine serum as a cell culture supplement for testing the toxicity of nanocarriers in non-bovine cell lines. Similarly, in case of in vivo studies, additional consideration may be required in translating the protein corona formed from mouse plasma to that from human plasma [108]. Furthermore, animal as well as human cells should be studied to determine whether species specificities exist.

- Unlike spherical nanocarriers, many diatom frustules and fragments exhibit disk, rod, or boat-like geometries with high aspect ratios and sharp edges. We hypothesize that these features could enhance margination towards vessel walls under flow potentially increasing endothelial interactions, promote non-uniform shear-induced rotation affecting adhesion and uptake, and lead to mechanical trapping in capillary beds (e.g., lung, spleen) for larger fragments. Therefore, shape-specific design principles such as disks for enhanced margination versus spheres for prolonged circulation could be strategically selected according to the application requirements.
- Currently, the in vivo fate of diatom-based nanocarriers is mainly studied in small rodents including mice and rats. Despite their promising biocompatibility in cell culture and small animals, comprehensive studies in large-animal models, particularly focusing on long-term biosafety, are critical to provide guidance for potential clinical trials. Therefore, several preclinical studies should be performed in nonhuman primates as well.
- It is more challenging to study the in vivo biodistribution, biodegradation and clearance of biosilica-based inorganic nanocarriers in comparison to organic nanoparticles. Therefore, advanced in vivo molecular imaging techniques and tools such as positron emission tomography (PET), magnetic resonance imaging (MRI), computed tomography (CT), and fluorescence imaging are needed to understand the in vivo interactions of diatom-based nanocarriers under physiological and pathological conditions following radiolabeling or fluorescent labeling [43]. For example, ^68^Ga radioisotope incorporated MSNs were employed for cell labeling, enabling whole-body single-cell tracking in mice using PET imaging [109].
- In published studies of diatom-based nanocarriers, the fluorescence markers/radioisotopes used were to specifically label cargo (e.g., drugs or bioimaging agents) or delivery system but not both. Therefore, it is important to study the co-localization of both diatom-based nanocarriers and their cargo using quantitative detection assays.
- The biodistribution of diatom-based nanocarriers should be studied in all organs and not just one. Only one study assessed the in vivo trafficking of these nanocarriers in all organs but reported only qualitative imaging information. Hence reporting of the quantitative data of such studies is suggested as it will be helpful to predict the potential safety of diatom-based nanocarriers.
- It is recommended to study the in vivo fate of diatom-based nanocarriers at both intracellular level as well as tissue/organ level.
- The diatom-based nanocarriers should be designed carefully keeping in mind their storage and transport requirements for clinical translation. Their design should support stability and resist temperature dependent physiochemical degradation pathways by selection of appropriate cryoprotectants.
- The in vivo fate of diatom-based nanocarriers should be studied not just until the delivery of their cargo but also after. It is of critical value to evaluate the pathways of their biodegradation and elimination and also their potential to be reused for physiological processes of organism.
- Finally, and importantly, integrating diatom-based nanocarriers alongside advances in computational modeling and artificial intelligence for predictive design, may further accelerate their optimization.

Diatom biosilica offers a rare combination of biological origin, structural complexity, and functional versatility. However, these same features introduce variables that complicate prediction of their in vivo behavior. The next phase of research should focus on turning structural diversity from a challenge into a design tool, enabling rational engineering of diatom-based nanocarriers with predictable and tunable biological outcomes. Looking ahead, the field must transition from descriptive studies to predictive frameworks by correlating frustule architecture with biological outcomes, ultimately enabling the rational design and clinical translation of diatom-based nanocarriers. From a design standpoint, future studies should aim to establish structure–function relationships that are specific to diatom biosilica. Testable hypotheses could include how frustule size, anisotropic and irregular geometry, and aspect ratio govern biodistribution and clearance pathways, whether hierarchical porosity alters immune recognition or macrophage uptake, and how species-specific architectures impact mechanical stability and degradation in physiological environments. Integrating advanced imaging, in vivo tracking, and computational modeling will be critical to disentangle these effects and to move toward predictive frameworks. Ultimately, establishing diatom biosilica as a uniquely programmable biomaterial could unlock new opportunities in targeted delivery, biosensing, and theranostics, while also addressing key translational challenges related to reproducibility, scalability, and safety.

In conclusion, this perspective integrates current evidence on the in vivo fate of diatom-based nanocarriers, highlighting key physicochemical and biological determinants while addressing critical challenges associated with long-term biosafety and clearance [10,110]. A major challenge for the field lies in bridging the gap between nanocarrier fate in controlled in vitro systems and the complex, heterogeneous in vivo environments, where diverse microenvironments, variable pH, protein coronas, and redox conditions complicate mechanistic understanding. Nevertheless, the successful translation of these engineered nanocarriers from in vitro models to clinical applications requires a comprehensive understanding of their pharmacokinetic profiles, including absorption, biodistribution, metabolism, and excretion in complex physiological environments [111]. Achieving an optimal balance between structural stability for effective cargo protection and biodegradability for safe elimination is essential for clinical success [39]. Notably the first-in-human clinical trials of Cornell silica dots developed by Wiesner and co-workers for targeting integrin-expressing cancers were successfully conducted using PET and optical molecular imaging [41]. These findings highlight the potential of silica and silica-based hybrid nanocarriers to enhance clinical outcomes and significantly improve patient care. Accordingly, this perspective advocates for systematic studies of the in vivo biodistribution and pharmacokinetics of diatom-based nanocarriers in small animals and nonhuman primates to enable the rational design of safer and more effective systems. Ultimately, bridging the gap between material design and biological performance, while addressing long-term safety and regulatory considerations, will be essential to unlock the full translational potential of diatom-based nanocarriers in precision medicine.

## Figures and Tables

**Table 2:** Key comparison between living biosilica and diatomaceous earth.

Characteristic	Living Biosilica	Diatomaceous Earth
Origin	Grown through active cultivation (fresh).	Obtained from fossils and minerals (ancient).
Purity	Very high, as produced in controlled environment and purified.	Low, contains inorganic impurities.
Composition	Is a monospecific culture.	Consists of mixed species.
Structure	Intact nanostructural integrity.	Often fragmented.
Morphology	Uniform with minimal variation in frustule size, shape, and porosity.	Heterogeneous (polydisperse) in frustule size, shape, and porosity.

## Data Availability

No new data were created or analyzed in this study. Data sharing is not applicable to this article.

## Abbreviations

The following abbreviations are used in this manuscript:

MSNs	Mesoporous silica nanoparticles
ROS	Reactive oxygen species
MTT	3-[4,5-dimethylthiazol-2-yl]-2,5-diphenyl tetrazolium bromide
PEG	Polyethylene glycol
CPP	Cell-penetrating peptide
PET	Positron emission tomography
MRI	Magnetic resonance imaging
CT	Computed tomography
PEI	Polyethyleneimine
DNPs	Diatomite nanoparticles
s.d.	Standard deviation
FDA	Food and Drug Administration
